# *In vivo* Visualization of Collagen Transdermal Absorption by Second-Harmonic Generation and Two-Photon Excited Fluorescence Microscopy

**DOI:** 10.3389/fchem.2022.925931

**Published:** 2022-06-03

**Authors:** Yanan Sun, Lishuang Li, Shuhua Ma, Gaiying He, Weifeng Yang, Yi Wang

**Affiliations:** ^1^ Experimental Research Center, China Academy of Chinese Medical Sciences, Beijing, China; ^2^ Beijing Key Laboratory of Research of Chinese Medicine on Prevention and Treatment for Major Disease, China Academy of Chinese Medical Sciences, Beijing, China

**Keywords:** transdermal absorption, second-harmonic generation, two-photon excited fluorescence, recombinant human collagen, live tracking

## Abstract

The transdermal administration of collagen is an important method used for wound healing and skin regeneration. However, due to the limitations of previous approaches, the process and degree of collagen transdermal absorption could only be quantitatively and qualitatively evaluated *in vitro*. In the present study, we introduced a novel approach that combines second-harmonic generation with two-photon excited fluorescence to visualize the dynamics of collagen transdermal absorption *in vivo*. High-resolution images showed that exogenous recombinant human collagen permeated the epidermis through hair follicles and sebaceous glands reached the dermis, and formed reticular structures in real time. We also validated these findings through traditional *in vitro* skin scanning and histological examination. Thus, our approach provides a reliable measurement for real-time evaluation of collagen absorption and treatment effects *in vivo*.

## 1 Introduction

The skin is the largest organ in the human body and plays a key role in protecting our body from the diverse external environment (Bouwstra et al., 2003). It is mainly composed of three layers that span from superficial to deep, including the epidermis, dermis, and hypodermis (Menon, 2015). The dermal skin contains components of the extracellular matrix, such as collagen fibers, that contribute to the strength and toughness of the skin (Ngan et al., 2015). As the main component of connective tissue, collagen is the most abundant protein in the skin, comprising 25–35% of the protein content in the whole body (Di Lullo et al., 2002). Collagen is also widely used in cosmetics and biomedical products (Toki et al., 2013; Sun et al., 2015). After exogenous collagen is smeared on the skin, it permeates through the cuticle to the dermis and forms a reticular structure to support the structure of the dermis and supply more extracellular matrix to keep the skin elastic (Chai et al., 2010). Thus, we need to dynamically visualize the process of transdermal absorption of exogenous collagen and measure the extent of collagen absorption with qualitative and quantitative approaches.

To date, various technologies have been applied to measure the biomedical administration of collagen to skin, including chemical assays (Fediuk et al., 2012), tissue inspection (Schulz et al., 2002), isotope tracing (Cross et al., 2007) and circulating diffusion chamber (Gamer et al., 2006), which can only be used to quantitatively examine the transdermal absorption of exogenous collagen *in vitro*. However, these approaches could not dynamically track the process of transdermal absorption *in vivo* and evaluate the real-time effects of biomedical administration. These limitations could be overcome by nonlinear optical microscopy, such as two-photon excitation fluorescence (TPEF) (Denk et al., 1990; König et al., 2007), and second harmonic generation (SHG) (Campagnola and Loew, 2003; Pittet et al., 2014). Among such technologies, SHG is a well-established method to directly visualize anisotropic biological tissues that possess large hyperpolarizabilities, such as collagen (Thrasivoulou et al., 2011). Moreover, SHG imaging has many advantages, such as a higher signal-to-noise ratio than autofluorescence imaging, detection of intrinsic signals without extrinsic dyes, and the utilization of nonlinear excitation from molecular fluorescence (Kiyomatsu et al., 2015). Nonetheless, few studies have examined the effects of drug administration on the skin using SHG because of the challenges in using it to distinguish endogenous from exogenous collagen. Recently, two-photon excited fluorescence (TPEF) imaging has been widely applied to visualize the dynamics of intrinsic molecules and cells in living specimens through conventional labeling via staining or fluorescent proteins (Miller et al., 2017). Due to the divergent mechanisms of SHG and TPEF, we speculate that they could be combined to provide complementary information for the dynamic observation of biological processes. However, few studies have reported the *in vivo* imaging of the transdermal absorption of collagen with a combination of SHG and TPEF (SHG-TPEF). Therefore, there is an urgent need to obtain *in vivo* high-resolution images of fibrillar collagen during the process of transdermal absorption using SHG-TPEF imaging, which could provide a novel approach for estimating the extent of drug absorption and further assessing the treatment effects of dermal drugs.

To address these issues, we first constructed a mouse model of transdermal absorption through administrating exogenous recombinant human collagen (R-hc), which was proved to be effective in skin absorption in our previous studies (Ma et al., 2018). Second, we set up the platform of *in vivo* SHG-TPEF skin imaging. Third, we tracked the dynamic process of collagen absorption of short-term administration by *in vivo* SHG-TPEF imaging and observed the transdermal absorption pathway. Finally, we applied Franz-type diffusion method to evaluate the collagen absorption at different time points as validation.

## 2 Methods

### 2.1 Animals

Healthy BALB/c mice were purchased from Vital River Laboratories (China). All animals used in this study were male adults (6–8 weeks, 20 ± 2 g body weight). Mice were kept in a specific pathogen-free environment with freely available food and water and a 12 h light-dark cycle. In this study, five mice were used at each time point, each of which received a topical dose of 1 mg/ml of R-hc on a shaved back on the left side of the thoracic spine and a saline control area on the right. The animal experiments were supervised and approved by the Research Ethics Committee of the China Academy of Chinese Medical Sciences ( ERCCACMS21-2106-12). All experiments compliance with the Regulations for the administration of affairs concerning experimental animals of the People’s Republic of China.

### 2.2 Quality Control of R-hc

#### 2.2.1.R-hc

R-hc is expressed by eukaryotic cells and integrates the human-like collagen gene, which is a heterozygous type I and type III collagen gene, into the yeast chromosome. The hydroxyproline in R-hc was replaced by proline. In our previous study, we analyzed the effect of R-hc on the repair of laser skin damage in mice. The results showed that R-hc can significantly accelerate wound healing, shorten wound healing time, and promote collagen production in wounds (Ma et al., 2018), which suggests that R-hc could be transdermally absorbed.

#### Fluorescent Label

For fluorescent labeling, a protein stock solution was prepared by mixing 100 mg R-hc freeze-dried powder (TianJin Irheaya Biological Technology Co., Ltd., Tianjin) with 10 ml 1 M phosphate buffer (pH 8.5–9.0) to produce the 1 ml protein labeling stock solution. To prepare the dye stock solution, anhydrous DMSO was added into a vial of iFluor™ 750 SE (AAT Bioquest, Ex/Em = 749/775 nm) to produce a 10–20 mM stock solution, which was mixed by vortexing. Then, 1 ml of the dye stock solution was added to the vial of protein solution (10 ml) and shaken thoroughly. The concentration of the protein was 10 mg/ml. The reaction mixture was rotated at 4°C for 12 h. The reaction mixture was loaded into a dialysis bag (<1 kd) and placed in a large beaker containing ultrapure water in the dark. The water was changed every 4 h until no fluorescence was detected in the ultrapure water. The fluorescent protein was freeze-dried into a powder for future use.

### 2.3 SHG-TPEF Skin Imaging

#### 2.3.1 In vivo

The back hair was shaved from the mice, which were treated with iFluor-R-hc (1 mg/ml) for 0, 1, 2, 3, 4 and 5 h at room temperature while avoiding light. At the end of the experiment, all mice were anesthetized with isoflurane and fixed on a mouse plate while the back skin was fixed on holder. The full-thickness skin tissue was scanned by a two-photon scanning confocal microscope (FV1000-MPE, Olympus, Japan).

A two-photon excited fluorescence microscopy system was used for image acquisition based on the reverse detection of SHG and two-photon excited fluorescence signals from the skin. The two-photon excited fluorescence microscopy system was equipped with a water-immersion objective lens (PLAN 25X OB. W IMM, NA 1.05, Olympus) and a Ti:sapphire laser oscillator (wavelength: 690–1040 nm; repetition rate: 80 MHz, pulse width: 100 fs; MaiTai HP DS-OL, Spectra-Physics, Inc. CA). The SHG image acquisition was performed at an excitation wavelength of 950 nm with a 690 nm dichroic mirror and a 475/20 nm emission filter (center wavelength/bandwidth). For the observation of iFluor-R-hc, an excitation wavelength of 790 nm and a 605–680 nm emission filter were used to isolate the SHG signal. The autofluorescence image was acquired with an excitation wavelength of 750 nm and a 495–540 nm emission filter.

The images were obtained from the epidermis and dermis and stored as a z-stack image sequence (step size of 1 μm in the z-axis). The image size was 512 × 512 pixels, and the scanning speed was 2 μm/pixel. The merging of the color channels was performed using FV1000 Viewer 3.1 software (Olympus). The three-dimensional image was performed using Imaris (Imaris ver. 8.4.2, Bitplane AG, Switzerland). To examine the collagen staining intensity, we calculated the SHG signal intensity, which has been considered a good predictor of collagen structure (Dunn et al., 2000; Theer et al., 2011). Thus, we calculated the signal intensity of SHG in the dermis at different time points after iFluor-R-hc administration and determined the collagen thickness based on the z-stack depth of the SHG signal.

#### 2.3.2 In vitro

Whole skin tissue (1 × 0.5 cm^2^) was removed at 0, 1, 2, 3, 4, and 5 h after the imaging of the mice. The tissues were examined as frozen sections (10 μm). The lateral plane of the skin was exposed under the objective lens of a two-photon excited fluorescence microscopy system. The above-mentioned scanning process was repeated to observe the iFluor-R-hc transdermal absorption. The laser condition settings were consistent with those used for the above-mentioned *in vivo* SHG-TPEF skin imaging.

### 2.4 HE, Immunofluorescence and Immunochemistry Staining

All frozen sections were subjected to hematoxylin and eosin (HE) staining for the histological study. Differential interference contrast images of the sections were captured using an Olympus BX51 microscope (PlanApo 20x/NA: 0.45).

The localization of collagen III and collagen I proteins was assessed by immunofluorescence. The skin tissue was taken from the left side of the thoracic spine after treated with R-hc for 24 h. The frozen sections were obtained in an ultralow temperature freezer after 30 min at room temperature. The slides were washed in 0.02 M PBS 3 times and then blocked with QuickBlock™ Blocking Buffer for Immunological Staining (Beyotime, China) for 1 h. A 1:500 dilution of anti-collagen III mouse monoclonal antibody and anti-collagen I rabbit polyclonal antibody (ab6310, ab34710; Abcam, United States) was added and incubated overnight at 4°C. After washing with PBS, the sections were incubated for 1 h at room temperature in the dark with a secondary antibody (Dylight 488-goat anti-mouse and Dylight 594-goat anti-rabbit; EARTHox, China). After immunostaining, the specimens were examined with a wide-field fluorescence microscope (BX51, Olympus, Tokyo, Japan) fitted with a digital camera (DP71; Olympus). The relative intensity of the staining was analyzed by Image Pro Plus 6.0 software (Media Cybernetics, Inc., United States).

The localization of R-hc was assessed by immunochemisty staining. The back of the rat was taken after treated with R-hc for 5 h. The protocol to follow was as follows: Endogenous peroxidase in the skin sections was blocked by hydrogen peroxide followed by a trypsin solution. After the protein block was used to eliminate background absorption, the samples were incubated with R-hc antibody ( Biogot technology, co, Ltd., China), followed by treatment with Rabbit streptavidin-biotin assay system (SP9001, Beijing Zhongshan Golden Bridge Biotechnology Co., Ltd., China). DAB staining solution was added to the sections. Hematoxylin was used for the nuclear counterstain. The slides were observed by Olympus microscope BX51.

### 2.5 SDS-PAGE and Western Blotting

The amount of total protein in the receptor solution was separated by sodium dodecyl sulfate-polyacrylamide gel electrophoresis (SDS-PAGE). The samples were stained with Coomassie Brilliant Blue R-250 stain (P0017F, Beyotime Biotechnology, China), and then scanned with ChemiDoc™ Touch Imaging System (Bio-Rad Laboratories, Inc., United States).

Protein samples were quantified using the Beyotime Protein Assay Kit (P0013, Nanjing, China). SDS/PAGE was used to separate molecules of different molecular weights. The proteins were transferred to PVDF membranes (Millipore, PVH00010) and then incubated with the R-hc antibody. Then an HRP-conjugated secondary antibody was added and the sample was incubated with an enhanced chemiluminescent reagent. GAPDH was used as an internal control, and scanning densitometry was applied to obtain protein signals.

### 2.6 The Franz-Type Diffusion

Mouse skin was mounted in the receptor compartment of a Franz-type diffusion cell (TP-6, HT, Jintao Instrument Technology Co., Ltd., Tianjin, China). Ten milligrams of iFluor-R-hc dissolved in normal saline or normal saline alone was added to the tight interface facing the mouse skin. The donor cap was covered with tin foil. A 0.9% sodium chloride buffer solution (pH 7.4) was used as the receptor solution. The receptor solution was stirred by a magnetic stirrer rotating at 300 rpm at 37°C. The samples were examined at 1, 2, 3, 4, and 6 h. The amount of total protein in the receptor solution was measured by a BCA protein assay kit (Beyotime, China), and the fluorescence intensity was measured with a fluorescence microplate reader (Synergy H1Hybrid Multi-Mode Reader, BioTek Instruments, Inc., United States) at Ex/Em = 640/670 nm.

### 2.7 Statistical Analysis

The results were statistically analyzed by one-way ANOVA and differences between groups were identified using Tukey’s post hoc test using GraphPad Prism v.6.0 (GraphPad software, Inc., La Jolla, Calif.). The difference was considered statistically significant at *p* < 0.05. Post-hoc analysis was performed with *p* < 0.05 as the threshold for statistical significance.

## 3 Results

### 3.1 R-hc Supplements Collagen in the Dermis by Transdermal Absorption

**FIGURE 1:**
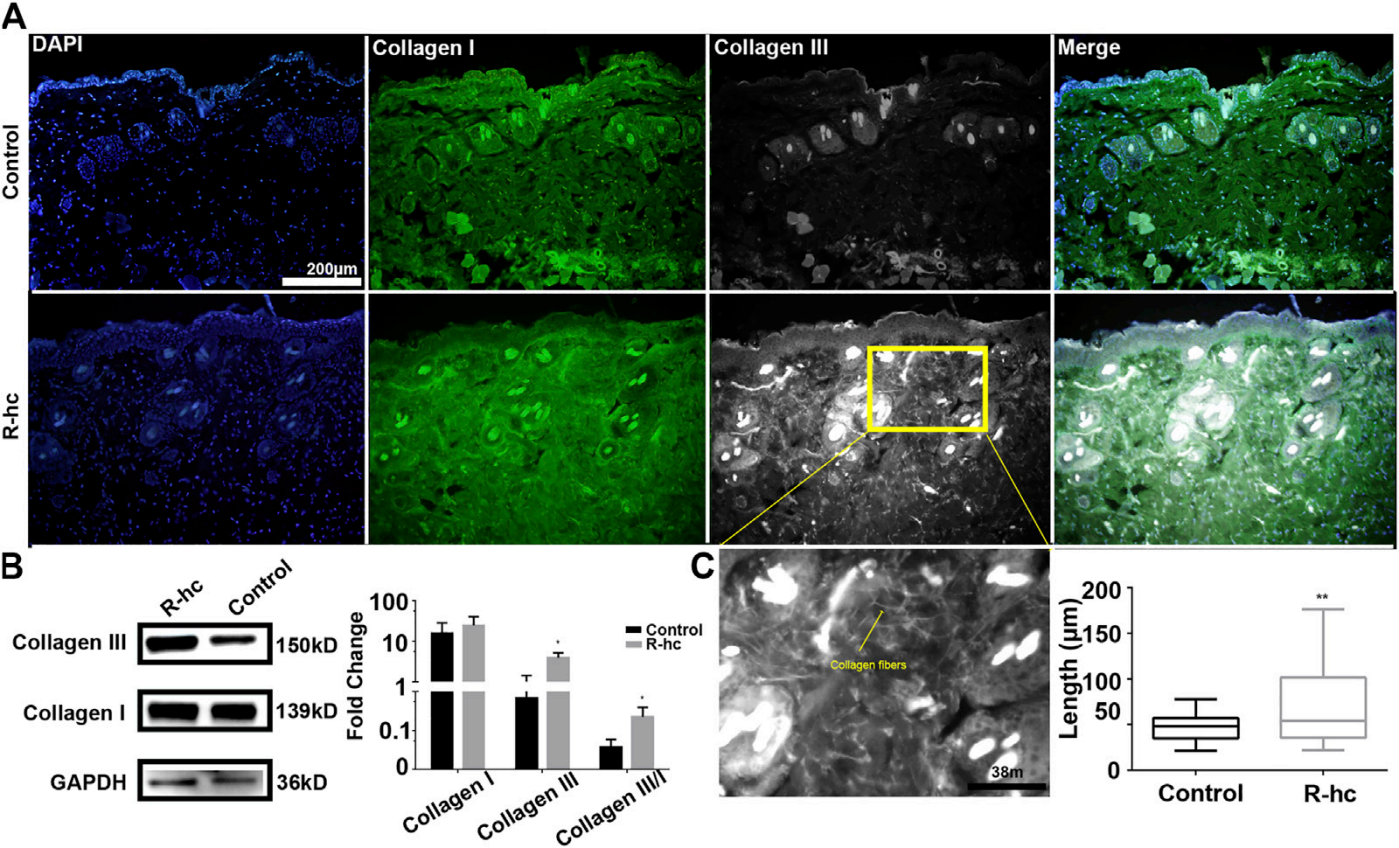
R-hc could be transdermally absorbed by the skin and increase the collagen content of the dermis. **(A)**. IF imaging of type I collagen (green), type Ⅲ collagen (white), and DAPI (blue); scale bar: 200 μm. **(B)**. Western blotting and quantitative results of collagen type I and Ⅲ (*n* = 3). Data are presented as means ± SD. *p < 0.05. **(C)**. Magnification image of the R-hc and quantitative results of collagen fibers, length, *n* = 10; scale bar: 38 μm. Data are presented as means ± SD. *p < 0.05, **p < 0.01 *vs*. Control.

24 h after R-hc administration, we observed an increase in the fluorescence intensity of collagen types I and III in the dermis relative to that observed in the control group (Figure 1A). Type III collagen was significantly increased by Western blotting (Figure 1B). At the same time, we calculated the ratio of type III and type I collagen (Figure 1B), and compared with the control group, the increase rate of type III collagen was more notable. We also observed that type III collagen was interwoven in the reticular structures in the superficial dermis (Figure 1C). Compared with the control group, the long fiber content of type III collagen increased significantly in the R-hc-treated group (Figure 1D). These findings suggest that R-hc could be transdermally absorbed and supplement type III collagen in the dermis.

### 3.2 Schematic Diagram of SHG-TPEF Skin Imaging

**FIGURE 2:**
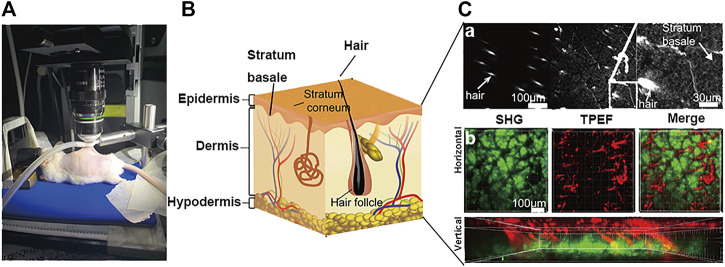
Schematic illustration of skin imaging. **(A)** *In vivo* SHG-TPEF skin imaging platform. **(B)**. Schematic diagram of the skin structure. **(C)**. Scanning images of different layers of the skin, including epidermis skin (a), dermis skin in 2D (horizontal plane) and 3D images (vertical plane) (b); scale bar: 100 μm.

We characterized the structure of the distribution of skin and iFluor-R-hc from the epidermis to the dermis. Specifically, in the epidermis, we used TPEF imaging at an excitation wavelength of 750 nm to observe the stratum spinosum and the stratum basale (Figure 2A). Then, in the dermis, we separately used SHG and TPEF imaging at excitation wavelengths of 950 and 790 nm, respectively, to visualize the dynamic process of iFluor-R-hc absorption (Figure 2B). Here, we used two-dimensional (2D) images and three-dimensional (3D) images to illustrate the scanning results. In the 2D images, we scanned the histological structures of the skin by utilizing the SHG signal (λ = 950 nm) and iFluor-R-hc by utilizing the TPEF signal (λ = 790 nm) (Figure 2B). Furthermore, as shown in Figure 2B, we revealed intrinsic collagen, and exogenous iFluor-R-hc was mutually linked to reticular structures in the dermis.

### 3.3 Tracking the Transdermal Absorption of iFluor-R-hc Using SHG-TPEF Imaging *in vivo*

**FIGURE 3:**
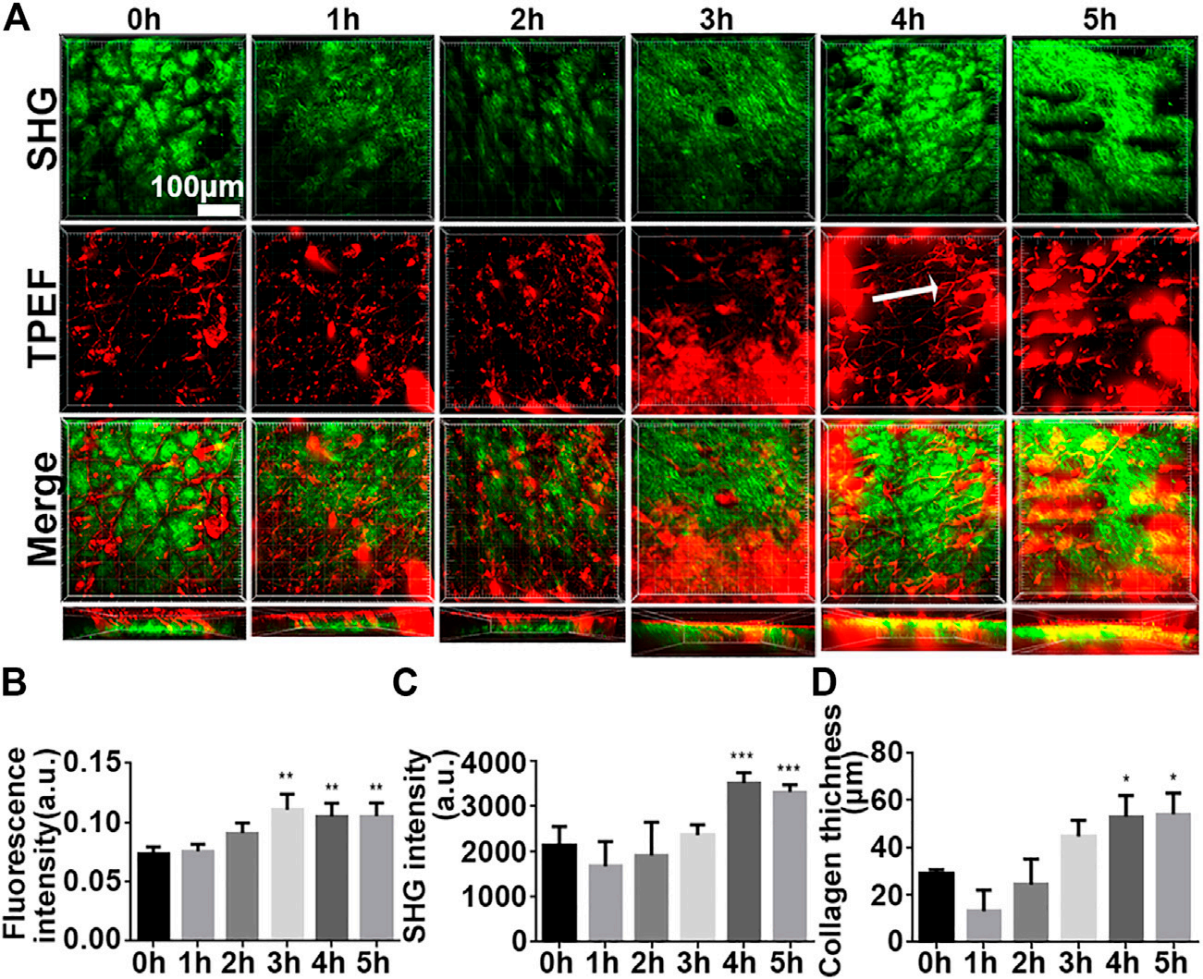
High-resolution images illustrated the process of iFluor-R-hc absorption by *in vivo* SHG-TPEF imaging. **(A)**. SHG image and TPEF image of iFluor-R-hc. White arrow represents reticular structure; scale bar: 100 μm. **(B)**. Statistical analysis of iFluor 750 nm intensity (*n* = 5). **(C)**. Statistical analysis of SHG intensity (*n* = 5). **(D)**. Statistical analysis of collagen layer thickness (n = 5). Data are presented as means ± SD. *p < 0.05, **p < 0.01, ***p < 0.001 *vs*. 0 h.

**FIGURE 4:**
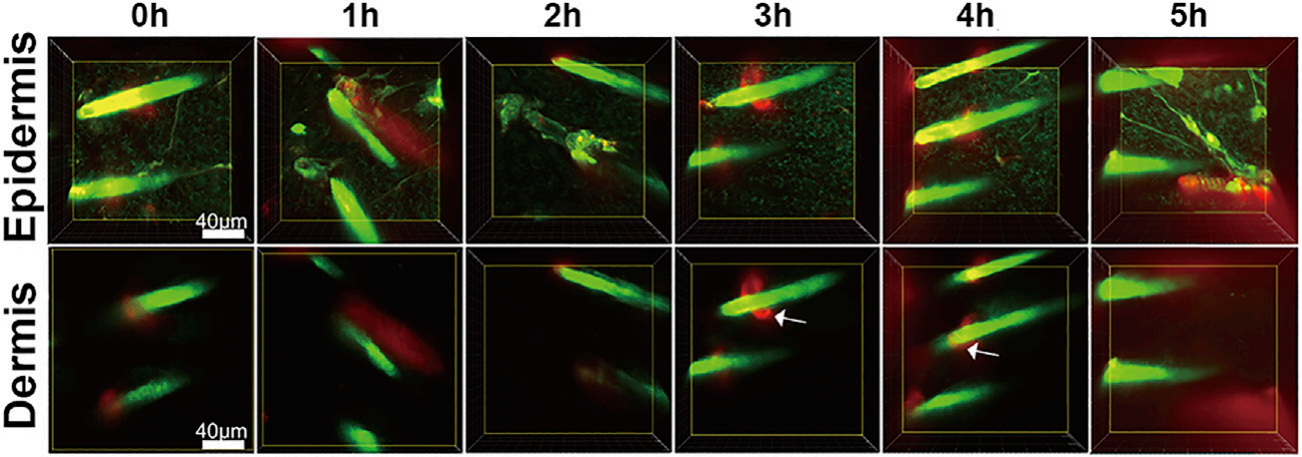
SHG-TPEF imaging of iFluor-R-hc around hair follicles. Green: hair shaft and stratum spinosum/stratum basale (λ = 750 nm); Red: iFluor-R-hc (λ = 790 nm); White arrow: iFluor-R-hc in hair follicle.

In this paper, the infrared fluorescent probe is used to label the to be detected, which can avoid autofluorescence of the skin, reducing background noise (Cross et al., 2007). We first analyzed mouse skin at the time point 0 h and showed a small amount of noise in the epidermis and autofluorescence of the dermis and TPE in the hair shaft (Figure 3A), which was considered to represent the baseline. Over time, we observed that the TPEF signal gradually increased (Figure 3A), suggesting that iFluor-R-hc permeated the cuticle and reached the dermis. Then, it formed reticular structures at the dermis-epidermal junction after 4 h (Figure 3A). Statistically, the iFluor intensity significantly increased after 3 h administration of iFluor-R-hc (Figure 3B), indicating that R-hc was gradually absorbed after 3 h of application. Furthermore, both the intensity of the SHG and the thickness of the collagen increased significantly after 4 and 5 h of iFluor-R-hc compared to baseline (Figures 3C,D, 4 h time point: t = 5.43, *p* < 0.001; 5 h time point: t = 4.68, *p* < 0.001), which indicated increased collagen density in the dermis.

To test whether our findings were the result of false positive signals in the *in vivo* SHG-TPEF imaging, we performed vertical plane observations. The results revealed that both iFluor and SHG intensity of iFluor-R-hc were significantly increased after 4 and 5 h iFluor-R-hc administration (Figures 4), which was consistent with the findings obtained from the *in vivo* SHG-TPEF imaging. The hair follicles were localized by histological examination; in the magnified TPEF image, the yellow arrows indicated the autofluorescence of the hair, while the white arrows in the circled follicles indicated the iFluor-R-hc absorbed by the hair follicles and sebaceous glands. This result indicated that R-HC may be absorbed into the dermis through the hair follicles and sebaceous glands.

### 3.4 Transdermal Absorption of R-HC Through Hair Follicles

**FIGURE 5:**
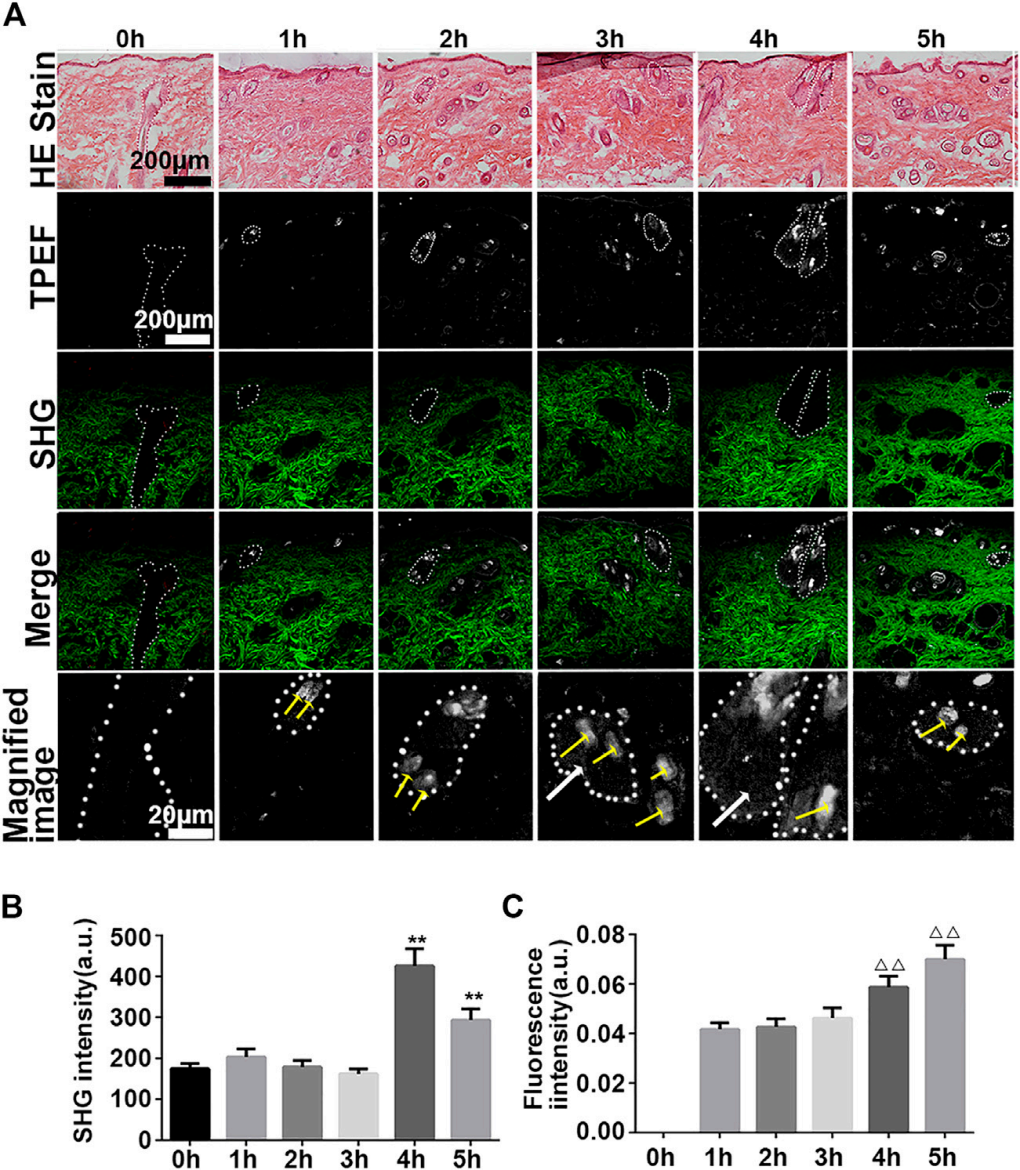
Vertical plane view of the skin after administration of iFluor-R-hc. **(A)**. HE imaging, SHG-TPEF imaging, and Magnified image of TPEF after iFluor-R-hc administration; scale bar: 100 μm, 20 μm (yellow arrows indicated hairs, white arrows indicated iFluor-R-hc, and white circles indicated hair follicles and sebaceous glands) **(B)** Statistical analysis of SHG intensity (*n* = 5). **(C)**. Statistical analysis of fluorescence intensity (*n* = 5). Data are presented as means ± SD. **p < 0.01 *vs*. 0h, ^△△^ *p* < 0.01 *vs.* 1 h.

We found that iFluor-R-hc could permeate and disperse around hair follicles after 3 h of administration (Figures 5B,C). This result is consistent with the results of our previous *in vitro* experiments that the protein first accumulated in the hair follicle and then absorbed into the dermis (Sun et al., 2017), which suggests that R-hc may be absorbed into the dermis through the hair follicle.

**FIGURE 6:**
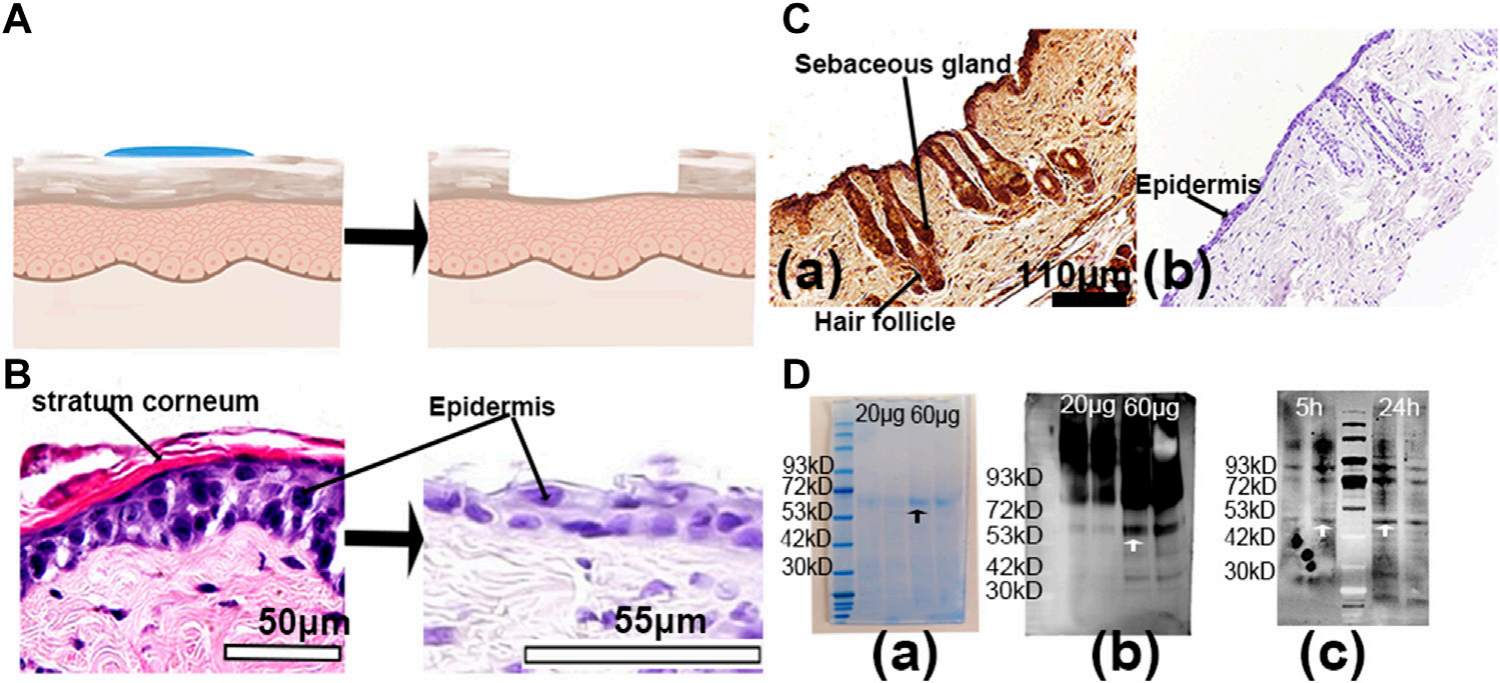
*In vitro* experiments of R-hc absorbed into the dermis through hair follicles. **(A)**. Schematic diagram of the tape stripping method. **(B)**. Skin tissue before and after tape stripping observed by histological staining; scale bar: 50 and 55 μm. **(C)**. Immunochemical staining of skin tissue with R-hc after 5 h of transdermal absorption, scale bar: 110um (a. with R-hc antibody, b. without R-hc antibody). **(D)**. Western blot of skin tissue (a. SDS-PAGE of R-hc, b. Western blot of R-hc, c. Western blot of administration of R-hc after 5 and 24 h.

To verify whether R-hc was absorbed into the dermis through the hair follicle, we removed the stratum corneum and part of the dermis by tape stripping (Figure 6A). After 20 times, the stratum corneum was removed and the epidermis was incomplete (Figure 6B). Immunohistochemical staining indicated that R-hc was localized in the epidermis, hair follicles, and sebaceous glands (Figures 6A–C), while no positive expression of R-hc was observed in images without the R-hc antibody (Figures 6B,C). The molecular weight of R-hc was around 55kD (Figures 6A–D). The results of the Western blot showed that the R-hc antibody could specifically immunoreact with R-hc (Figures 6B–D). The skin tissue was taken out of R-hc after 5 and 24 h of transdermal absorption, the stratum corneum and part of the epidermis were removed, and the R-hc in the skin was detected by Western blotting. A clear band near 55kd was observed.

### *3.5 In vitro* Tracking of the Transdermal Absorption of iFluor-R-hc

**FIGURE 7:**
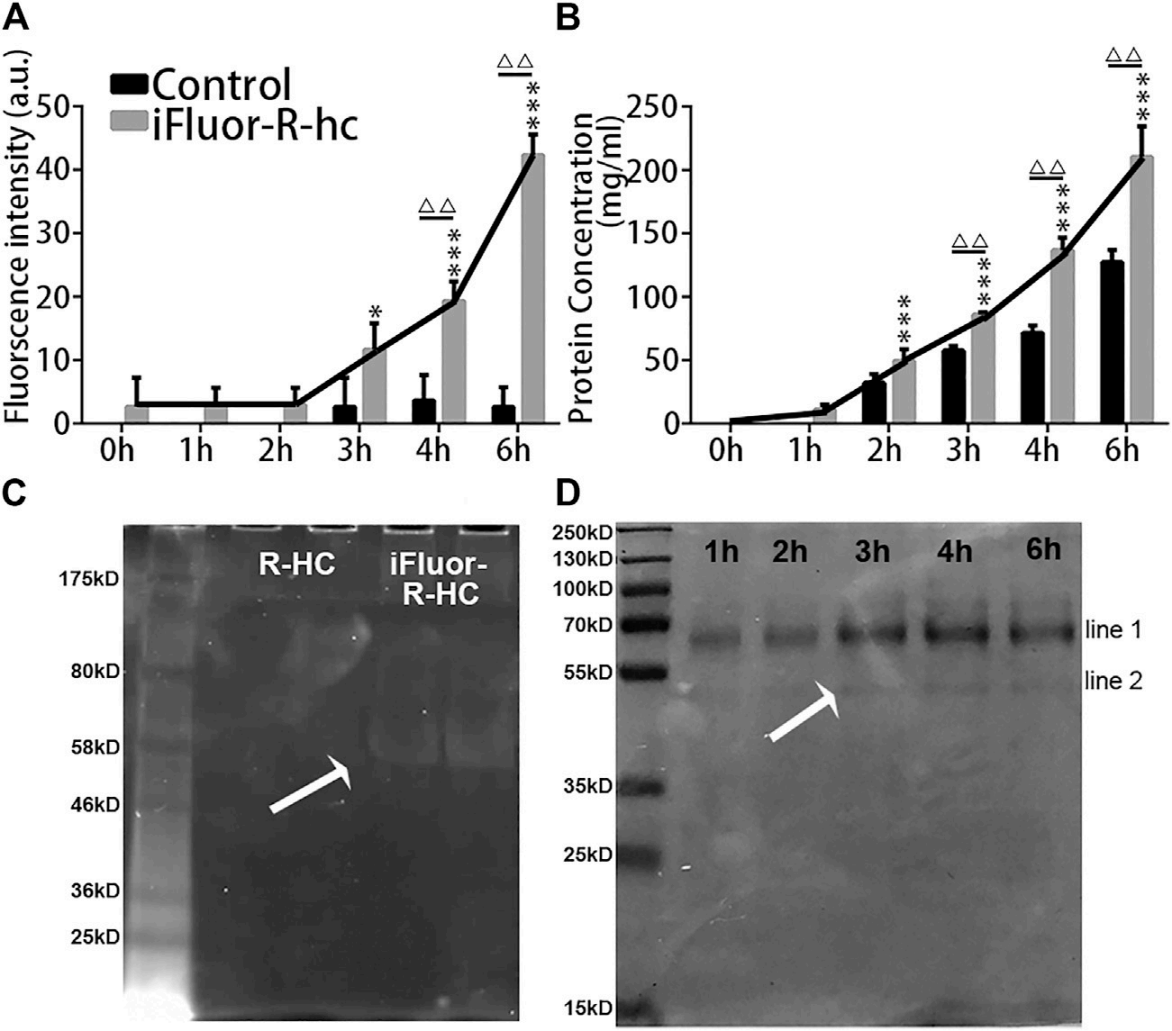
*In vitro* examination of iFluor-R-hc Transdermal absorption. **(A)**. iFluor-R-hc fluorescence intensity detected by fluorescence microplate reader (*n* = 5). **(B)**. Determination of iFluor-R-hc concentration by BCA method (*n* = 5). **(C)**. R-hc was labeled with iFluor™ 750 SE, Ex = 700 nm. The molecular weight of R-hc is between 46 kd and 58 kd. **(D)**. Coomassie blue staining of iFluor-R-hc. The protein band shown in line1 was between 55 kd and 70 kd, which may be a water-soluble protein degraded in skin tissue during transdermal absorption. The protein band shown in line2 was close to the molecular weight at around 55 kd. Data are presented as means ± SD.*p < 0.05, ***p < 0.01 *vs.* 1h; ^△△^ *p* < 0.01 *vs.* Control.

Next, we used traditional *in vitro* Franz diffusion cells to test whether *in vivo* SHG-TPEF imaging could sensitively and accurately track transdermal absorption, which could confirm the generality of our novel approach. Specifically, compared with the baseline (0 h), we first observed the significantly increased fluorescence intensity of iFluor-R-hc after 3 h of administration (Figure 7A). Moreover, the iFluor-R-hc group showed significantly increased protein concentrations after 2 h of administration of iFluor-R-hc (Figure 7B). Compared with the control group, the iFluor-R-hc group showed both significantly increased iFluor intensity and iFluor-R-hc concentration after 4 h of administration of iFluor-R-hc (Figures 7A,B). These findings were consistent with the observations of *in vivo* SHG-TPEF imaging, which suggests that our novel approach could sensitively and dynamically visualize collagen transdermal absorption *in vivo*. In addition, in the collected liquid, we found no change in the molecular weight of R-hc after iFluor™ 750 SE labeling, and there was a clear target protein band about 55 kD after 4 h of administration in the iFluor-R-hc group (Figures 7C,D).

## 4 Discussion

In this study, we used a novel noninvasive approach to dynamically capture the process of collagen transdermal absorption *in vivo* by combining SHG with TPEF. Both two-photon fluorescence imaging and second harmonic imaging are second-order nonlinear processes. In addition to the needs of different filter addition, TPEF and SHG are almost identical in the optical detection system, the obtained information for both complement and confirm each other, so it has received much attention in recent years. In this paper, the infrared fluorescent probe was used to label the collagen, which can avoid the autofluorescence of the skin and reducing background noise (Li et al., 2019). Over time, we observed that collagen permeated through the hair follicle, reached the dermis, and formed reticular structures in the dermis after 4 h of administration of exogenous iFluor-R-hc, which was also confirmed by *in vitro* examinations. To the best of our knowledge, this approach is the first to be used for the *in vivo* visualization of the transdermal absorption of biochemically administered collagen, and these findings provide a technological method for the clinical assessment of transdermal drugs in the future.

A previous study showed that compounds with a molecular weight greater than 50 kDa penetrate the dermis was difficulty because of blockage by the stratum corneum (Ogiso et al., 2000). Therefore, various transdermal drug delivery systems have been developed (Chang et al., 2019; Lin et al., 2021), but whether macromolecules can penetrate the skin is still not clear. In our study, we used *in vivo* SHG-TPEF skin imaging to track the process of transdermal absorption of exogenous collagen, which is approximately 55 kDa in size. The iFluor fluorescence signal was first detected in the hair follicle 3 h after coating R-hc on the skin, which confirms that macromolecules could be absorbed by the skin. The above experimental results were confirmed by immunohistochemical staining and western blotting. Moreover, this combined technology has many advantages, such as noninvasiveness, the absence of conventional dyes, and good penetration of biological tissues (Kiyomatsu et al., 2015). Thus, it allows the long-term observation of dynamic changes at the same skin site, which can be extended to the development of an *in vivo* real-time assessment method for cosmetics and biomedical administration.

The results of the examination of R-hc transdermal absorption provided a quantitative approach for the *in vivo* assessment of the transdermal absorption of protein drugs through the combined measurement of collagen density and absorption. Specifically, we first demonstrated that a typical application of R-hc increased type III collagen and collagen fiber growth, which suggests that R-hc could increase skin elasticity. Second, we used TPEF combined with SHG imaging to study the manner and depth of R-hc absorption *in vivo*. Since the stratum corneum acts as a physical barrier to skin on the surface of the epidermis (Elias and Choi, 2005), it has an impact on the penetration efficiency of R-hc. However, the results of the *in vivo* transdermal absorption model showed that the fluorescence intensity of type III collagen was significantly increased after 24 h of R-hc administration, which suggesed that iFluor-R-hc could still effectively permeate the epidermal skin. Potts and colleagues found that an increased molecular weight increased lipophilicity and permeability, which suggests that the penetration of drugs into the skin is tightly associated with the molecular characteristics of the drug (Potts and Guy, 1992). Lin and colleagues also considered that the effect of lipophilicity is a more dominant factor than the molecular weight in the passive diffusion permeability (Lin et al., 1996). We observed the fluorescence signal of iFluor in the hair follicle after 3 h of R-hc application by *in vivo* SHG-TPEF imaging, and the dermis was interlaced into a reticular network after 4 h. Therefore, we assume that the osmotic effect of R-hc in the dermis could be attributed to higher permeability.

HE staining is a classic method to detect the pathophysiological changes of skin, but it can only observe the skin’s thickness changes in various layers, inflammatory cell invasion, and other indicators, but the absorption and distribution route and collagen content, which are directly related to wound healing or drug efficacy assessment, can not be observed. In our study, we examined SHG intensity to measure the content of collagen in the dermal skin. SHG imaging provides increased resolution and contrast when examining collagen than typical histological staining analysis (Tanaka et al., 2013). Moreover, it is also a feasible and fast method that could be applied to the imaging of thicker tissue without fixing, slicing and staining (Sun et al., 2003). SHG intensity could accurately reflect the *in vivo* changes in collagen. Specifically, in the examination of HE staining of R-hc, we found that the amount of R-hc gradually increased after iFluor-R-hc administration and reached a peak at the 4 h time point. Similar results were also observed in the analysis of Franz-type diffusion, which was consistent with the findings of *in vivo* SHG-TPEF skin imaging. These findings indicate that SHG imaging could provide reliable measurements for the real-time evaluation of collagen absorption.

Collagen is one of the core constituents that maintains the elasticity of the skin; thus, reducing collagen in the skin would result in wrinkled and flabby skin (Lee et al., 2001). In our study, we observed that R-hc gradually permeated the dermal skin and formed the reticular structure after 3 h of administration. This suggests that exogenous collagen could not only supplement skin collagen to meet the needs of anti-aging cosmetics but also form granulation tissue together with intrinsic collagen to fill wounds in tissue.

SHG-TPEF imaging allows us to observe the manner of transdermal absorption and aggregation sites of R-hc *in vivo*, but some limitations still exist for this technology. For example, individual differences in cortical thickness might cause variation in the scanning results, which could have an impact on examining SHG intensity. Alternatively, the autofluorescence of tissue might affect the detection of exogenous collagen labeled with fluorescent probes. A previous study showed that the excitation wavelength of tissue autofluorescence was between 720 and 880 nm, and the overall signal sensitivity (420–490 nm) was affected by the convolution of the spectral transmittance and the detector efficiency (Breunig et al., 2010). Therefore, the use of infrared dyes could be useful for the fluorescent labeling of exogenous proteins, and bandpass filters could be adjusted to reduce the effects of autofluorescence on skin scanning.

**FIGURE 8:**
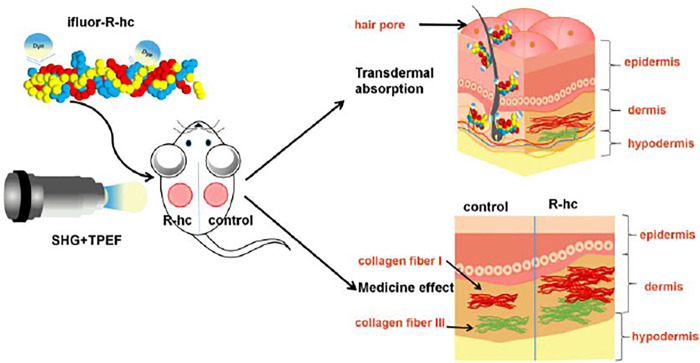
Schematic representation of the transdermal absorption of R-hc. The R-hc was fluorescently labeled and smeared on the back of the mice. The dynamic process of R-hc transdermal absorption was observed by SHG-TPEF imaging *in vivo*. R-hc can be absorbed into the dermis through hair follicles and sebaceous glands, and increases the content of type III collagen in the dermis.

In this study, we used *in vivo* SHG-TPEF skin imaging to track the dynamic process of the transdermal absorption of exogenous collagen in a mouse model. We observed that exogenous collagen gradually permeated the dermal skin via hair follicles and sebaceous glands and formed a reticular structure (Figure 8), which was also confirmed by traditional *in vitro* skin imaging. This is the first study to use a noninvasive approach to visualize the absorption of exogenous collagen in skin *in vivo*, which could extend our understanding of the process of skin absorption and provide the feasible technology for the clinical assessment of drugs for external use.

## Data Availability

The original contributions presented in the study are included in the article/Supplementary Material; further inquiries can be directed to the corresponding author.
